# Microfluidics Technology in SARS-CoV-2 Diagnosis and Beyond: A Systematic Review

**DOI:** 10.3390/life12050649

**Published:** 2022-04-27

**Authors:** Mohd. Raeed Jamiruddin, Bushra Ayat Meghla, Dewan Zubaer Islam, Taslima Akter Tisha, Shahad Saif Khandker, Mohib Ullah Khondoker, Md. Ahsanul Haq, Nihad Adnan, Mainul Haque

**Affiliations:** 1 Department of Pharmacy, BRAC University, Dhaka 1212, Bangladesh; mohd.raeed@bracu.ac.bd; 2 Department of Microbiology, Jahangirnagar University, Savar, Dhaka 1342, Bangladesh; meghla.ju@gmail.com (B.A.M.); dewanzubaerju@gmail.com (D.Z.I.); taslima.tisha.bd@gmail.com (T.A.T.); 3 Gonoshasthaya-RNA Molecular Diagnostic & Research Center, Dhanmondi, Dhaka 1205, Bangladesh; shahad@rnabiotech.com.bd (S.S.K.); ahsan@rnabiotech.com.bd (M.A.H.); 4 Department of Community Medicine, Gonoshasthaya Samaj Vittik Medical College, Savar, Dhaka 1344, Bangladesh; mohib@gonoshasthayakendra.org; 5 The Unit of Pharmacology, Faculty of Medicine and Defence Health, Universiti Pertahanan Nasional Malaysia (National Defence University of Malaysia), Kem Perdana Sugai Besi, Kuala Lumpur 57000, Malaysia

**Keywords:** microfluidics, SARS-CoV-2, COVID-19, antibody, antigen, nucleic acid

## Abstract

With the progression of the COVID-19 pandemic, new technologies are being implemented for more rapid, scalable, and sensitive diagnostics. The implementation of microfluidic techniques and their amalgamation with different detection techniques has led to innovative diagnostics kits to detect SARS-CoV-2 antibodies, antigens, and nucleic acids. In this review, we explore the different microfluidic-based diagnostics kits and how their amalgamation with the various detection techniques has spearheaded their availability throughout the world. Three other online databases, PubMed, ScienceDirect, and Google Scholar, were referred for articles. One thousand one hundred sixty-four articles were determined with the search algorithm of microfluidics followed by diagnostics and SARS-CoV-2. We found that most of the materials used to produce microfluidics devices were the polymer materials such as PDMS, PMMA, and others. Centrifugal force is the most commonly used fluid manipulation technique, followed by electrochemical pumping, capillary action, and isotachophoresis. The implementation of the detection technique varied. In the case of antibody detection, spectrometer-based detection was most common, followed by fluorescence-based as well as colorimetry-based. In contrast, antigen detection implemented electrochemical-based detection followed by fluorescence-based detection, and spectrometer-based detection were most common. Finally, nucleic acid detection exclusively implements fluorescence-based detection with a few colorimetry-based detections. It has been further observed that the sensitivity and specificity of most devices varied with implementing the detection-based technique alongside the fluid manipulation technique. Most microfluidics devices are simple and incorporate the detection-based system within the device. This simplifies the deployment of such devices in a wide range of environments. They can play a significant role in increasing the rate of infection detection and facilitating better health services.

## 1. Introduction

Coronavirus disease-19 (COVID-19) and other infectious diseases are a significant concern worldwide. During pandemics, early diagnosis and rapid identification of people with the disease are critical for treating infected patients and controlling disease spread. The World Health Organization (WHO), as well as the US Centers for Disease Control and Prevention (CDC), developed and published protocols for reverse-transcriptase polymeric chain reaction (RT-PCR) as a frontline diagnostic tool for COVID-19. Though the tests are highly specific and sensitive, they are designed for implementation in large centralized diagnostic laboratories due to their time-consuming as well as labor-intensive nature [1,2]. The continued global spread of COVID-19 demonstrates significant gaps in our ability to respond to new virulent pathogens, which can be addressed by implementing rapid, accurate, and easily configurable molecular diagnostic tests.

Microfluidics describes systems that manipulate and analyze small volumes of fluids [3]. The technology incorporates tools to manipulate small volumes of fluids for controlling chemical, biological, and physical processes [4]. Microfluidics devices have been prepared and studied for the last 15 years [5]. However, its application in the biological field gained traction in recent decades. Such devices have been termed “Lab-on-a-chip” [5]. Silicon substrates were used to design microfluidic devices during the 1980s and 1990s [6]. Development and designing of such devices required cleanroom facility and strong know-how, which hindered its wide range of adoption [6]. However, with the advent of the new millennia, the use of polymer-based microfabrication and elastomer casting allowed for mass production of such devices outside the confines of a cleanroom [7,8]. The most common polymer used was found to be polydimethylsiloxane (PDMS), followed by thermoplastics as well as thermosets [9]. Other than glass, silicon, metal, printed circuit boards (PCBs), and others have fallen in use due to the complexity in their preparation [9,10]. In addition to being inert and rigid, glass also provides the added advantage of a transparent surface on which optical methods can be incorporated [9].

Microfluidics techniques are primarily dependent on diffusion. However, the scale factor can predominate certain factors such as laminar flow, decrease in diffusion time, and fast response to microsystem changes. Two main types of fluid manipulation implemented in microfluidics are continuous flow microfluidics and segmented flow microfluidics. The latter’s significance over the former is the elimination of hydrodynamic dispersion and the generation of the isolated reaction vessel [10]. This reduces the need for substantial reagents and relatively long channel lengths [11].

Over the years, microfluidics technology and nanotechnology have been incorporated into the development of diagnostic tools. Complex microfluidic devices have been designed to include a quantitative PCR (qPCR) system in which channels carry the substrates through the necessary temperature gradient resulting in denaturation, then annealing, and finally elongation. The number of channels incorporated within the device represents the number of PCR replication [12,13].

Recent developments in microfluidic technology have emerged as a powerful tool for improving methods of individually tailored disease diagnosis and treatment [14]. Compared to traditional methods, microfluidic devices require a much smaller volume of biological samples for testing disease biomarkers in a brief period. Furthermore, using a multichannel detection technique, parallel assays analysis based on a single microfluidic device can be easily accomplished, providing statistically meaningful data for analysis [15,16]. Moreover, using microfluidic devices to create an individualized care plan will enable one to accurately and quickly control and program drug delivery profiles customized with a set of drug administration approaches for each particular patient [17].

**Figure 1:**
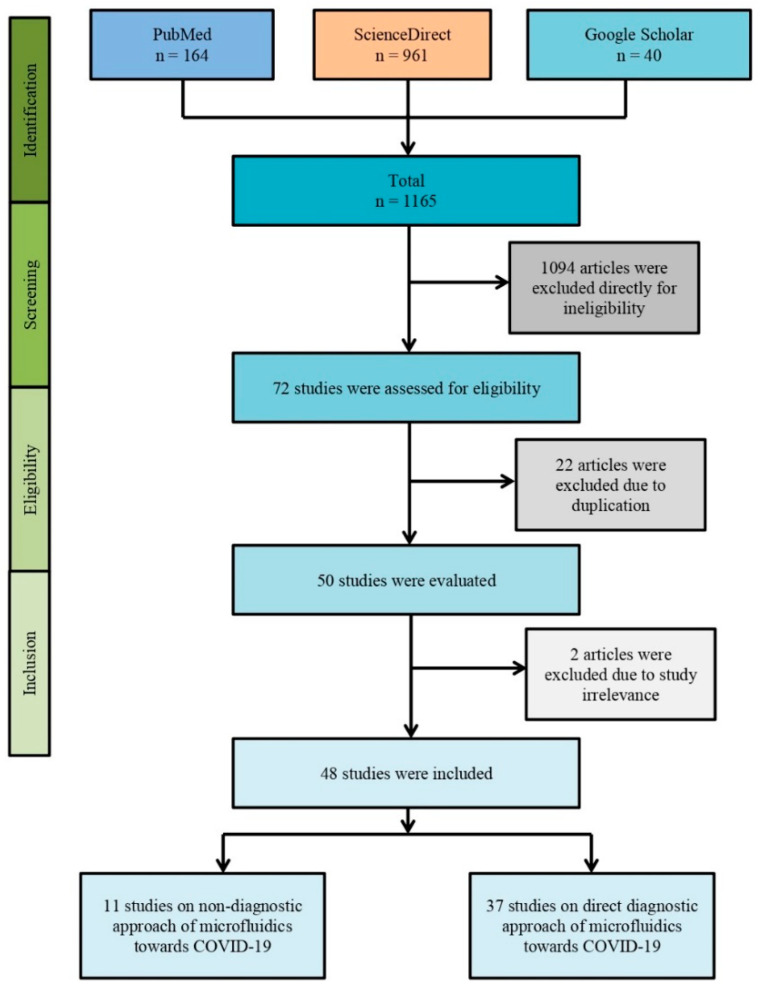
The PRISMA flow diagram of the methodology where initially 1165 articles were found with our search strategy from different online databases such as PubMed, ScienceDirect, and Google Scholar. Overall, 1094 articles were excluded as they were not original research articles but rather case reports, review articles, correspondence, or letters. Of the 71 articles, 22 articles were excluded due to study duplication. Eventually, 48 articles were included in this systematic review after excluding pre-prints (n = 2).

This review discussed multiple microfluidic devices integrated with various assays that detect severe acute respiratory syndrome–coronavirus-2 (SARS-CoV-2) whole virus, antigen, anti-SARS-CoV-2 antibodies, or nucleic acid (Figure 1). Overall, these developed kits enable low-cost, rapid, and precise detection with high sensitivity and specificity. They are suitable for application in resource-poor settings as point-of-care testing (POCT). There is no alternative to rapid testing and isolating infected individuals to control the rapid spread of the novel coronavirus. These microfluidic rapid test kits can play a vital role in this purpose. Furthermore, integrated microfluidic devices have other applications such as diagnosing COVID-19 disease progression, whole-genome sequencing of SARS-CoV-2, and angiotensin-converting enzyme 2 (ACE-2) engineered microfluidic microspheres for intratracheal neutralization of virus, etc. So, microfluidic technology can be used in various ways to control this current growing pandemic.

## 2. Methodology

### 2.1. Search Strategy

**Figure 2:**
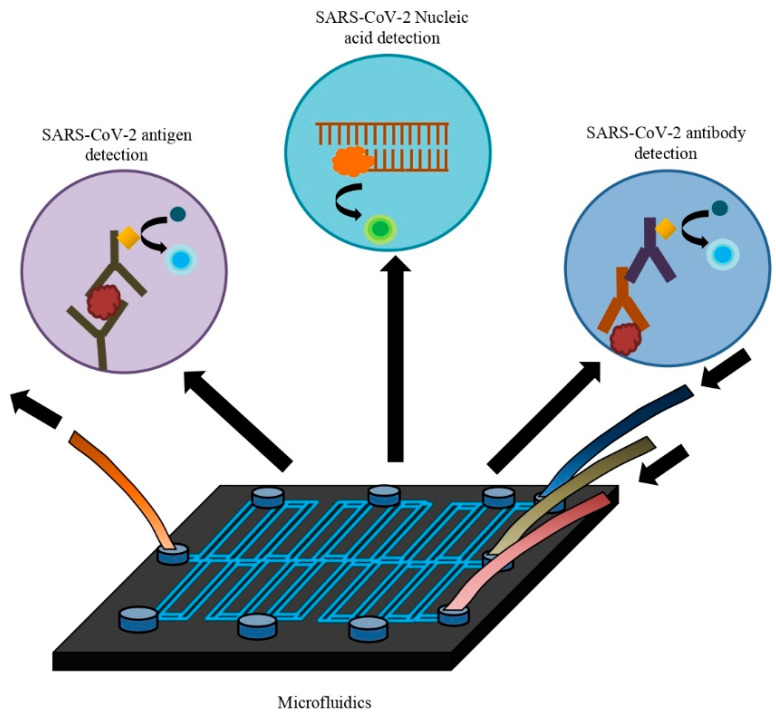
Microfluidics in the diagnosis of SARS-CoV-2 nucleic acid, antigen, and antibody.

Following Preferred Reporting Items for Systematic Reviews and Meta-Analyses (PRISMA) guidelines, a proper search strategy was maintained. Three individual databases (i.e., Google Scholar, PubMed, and ScienceDirect) were assessed rigorously to find original articles. Specific keywords such as “microfluidic”, “microfluidics”, “SARS-CoV-2”, “COVID-19”, and “novel coronavirus” with Boolean logical operators (i.e., “AND” and “OR”) were used to search the articles. The detail is demonstrated in Figure 2.

### 2.2. Eligibility Criteria

For this systematic review, only published original articles were included. Articles other than original studies such as reviews, case studies, editorials, letters to the editor, comments, and correspondence were excluded. The search was conducted until 25 January 2022 with no year restriction. Only English articles were included. The duplications of articles were removed carefully. The references were handled using EndNote software (version X9, Clarivate, Philadelphia, PA, USA).

### 2.3. Study Assessment and Inclusion

After searching the databases, the authors respectively assessed and selected for this systematic review based on the eligibility criteria. After the individual assessment and inclusion of the selected articles, the authors took part in further discussion to resolve any confusion, issues, errors, or biases regarding the selected articles.

## 3. Microfluidics in SARS-CoV-2

### 3.1. Microfluidics in SARS-CoV-2 Antibody Diagnosis

#### 3.1.1. Fluorescence-Based Detection for Antibody Diagnosis

SARS-CoV-2 antibody detection is crucial for retrospective sero-surveillance and the assessment of vaccine efficacy [18,19,20]. Microfluidics uses different techniques to detect SARS-CoV-2 antibodies, such as the microfluidic DA-D4 (double-antigen bridging immunoassay technique, which detects total antibodies including all subclasses and isotypes) based technique developed by Heggestad et al., and sandwich/competitive immune-sensors based techniques developed by Lin et al., which reports a run capacity of 24 and three samples per device, respectively. The semi-automated microfluidic platform with the classic multilayer soft-lithography technique developed by Monkayo et al. can detect antibodies against four SARS-CoV-2 antigens while running 50 samples in a single device. The mechanically induced trapping of molecular interactions (MITOMI) based microfluidic nano-immunoassay developed by Swank et al. reported the highest sample running capacity (1024 samples per device) (Table 1) [21,22,23,24].

The MITOMI-based microfluidic technique requires the least amount of sample (0.6 µL only) for antibody detection as well, compared to the semi-automated multiplexed microfluidic technique (6 µL), sandwich/competitive immune-sensors based technique (10 µL), nano-interstices, and digitized flow control-based technique (10 µL), and the DA-D4-based microfluidic technique (60 µL) (Table 1) [21,22,24,25].

The semi-automated multiplexed microfluidic technique and the DA-D4-based method can detect antibodies against Spike (S), S1 protein, nucleocapsid (N), and RBD antigens. On the contrary, MITOMI-based nano-immunoassay, sandwich/competitive immune-sensors-based technique, and the nano-interstice-based technique can detect antibodies against only S, RBD, and N, respectively (Table 1) [21,22,23,24].

Whereas the MITOMI-based nano-immunoassay technique and DA-D4-based methods can detect antibodies by directly processing blood samples, the three other fluorescent-based detection techniques require serum samples to be loaded [21,24]. Swank et al. reported a low-cost ultra-flow volume whole-blood sampling method along with his MITOMI-based detection technique that can collect small amounts of blood samples by a single finger prick and thus eliminate the need for venipuncture [24]. This facility is absent in other microfluidic-based techniques stated above.

The microfluidic serological assay combining nano-interstices and digitized flow control is capable of detecting anti-N IgG and IgM in only 5 min with a sensitivity and specificity of 91.67% and 100% validated by testing 152 serum samples as described by Lee et al. However, the semi-automated multiplexed microfluidic technique takes a comparatively longer time (2.6 h) to detect antibodies with a sensitivity of 95% and specificity of 91% based on testing serum samples collected from 66 COVID-19 patients [23,25]. The MITOMI-based nano-immunoassay showed specificity and sensitivity of 100% and 98%, respectively, upon analyzing 289 serum samples [24]. Heggestad et al. reported a specificity of 100% and a sensitivity of 100% for anti-S1 and anti-RBD and 96.3% for anti-N antibodies validated by testing 46 positive and 41 negative plasma samples (Table 1) [21].

#### 3.1.2. Spectrometer and Image Analysis-Based Detection

The automated enzyme-linked immunosorbent assay (ELISA) on-chip microfluidic platform developed by Gonzalez-Gonzalez et al., the Opto microfluidic sensing platform based on localizes surface plasmon resonance (LSPR) developed by Funari et al., paper-based pulling force spinning top microfluidic devices designed by Gong et al., microfluidic chemiluminescent ELISA technique by Tan et al., and the reciprocating flowing immunobinding strategy by Liu et al. detect anti-SARS-CoV-2 antibodies by spectrometer-based detection techniques (Table 1) [26,27,28,29,30].

Among these techniques, the paper-based pulling force spinning top microfluidic devices can detect anti-RBD IgG, IgM, or IgA. In contrast, other spectrometer-based detection techniques stated above can detect anti-S or anti-N IgG. This technique has another advantage in that it can directly collect serum from blood samples by pulling force spinning top technique within 4–5 min and detect the IgA, IgM, and IgG with high sensitivity (i.e., 97.1% for IgA, 91.4% for IgM, and 85.7% for IgG) and specificity (i.e., 100% for IgA, 92.8% for IgM, and 100% for IgG) by measuring various combinations of these antibodies as described by Gong et al. [27]. The other spectrometer-based detection techniques rely on either serum or plasma samples.

The microfluidic-based on-chip ELISA techniques show their limitations in cases of efficient immunobinding, being designed with a flow-through flowing technique that directs the antibody-containing serum solution in one direction towards the pre-coated antigens for quick immunobinding. To reduce this limitation, Liu et al. developed a reciprocating-flow ELISA technique that integrates a pressure regulating modular (a pure water bottle) which controls the flow of the antibody-containing fluid through the antigen (i.e., SARS-CoV-2 N protein) pre-coated site forward and backward repeatedly by exerting a controllable pressure to increase the probability of collisions among antibodies and antigens which eventually decrease the time required for adequate immunobinding. This technique has reduced the time for anti-N IgG detection to less than 5 min. Another advantage of this technique is that the colorimetric reactions can be photographed with a commercial smartphone and analyzed by using software (ImageJ) with a higher LOD of 4.14 pg/mL compared to the other ELISA on-chip platform as the Opto microfluidic sensing platform (LOD: 0.08 ng/mL) and microfluidic chemiluminescent ELISA (LOD: 10 pg/mL) [26,29].

Gonzalez-Gonzalez et al., Liu et al., and Gong et al. reported the use of commercial smartphone cameras to take photographs of the resulting colorimetric reactions and analyze the images with ImageJ software to detect and quantify the antibody concentrations with a high limit of detections. In contrast, the Opto microfluidic sensing platform developed by Funari et al. and the microfluidic chemiluminescent ELISA-based platform designed by Tan et al. require comparatively complex and extensive analyzing equipment NanoDrop One UV-Vis spectrometer (Thermo Fisher’s, USA) and CMOS camera along with ImageJ software (NIH, Bethesda, MD, USA) [26,27,28,29,30].

All the spectrometer based microfluidic platforms that function on the principles of the ELISA technique are capable of detecting SARS-CoV-2 specific antibodies in serum or plasma samples with minimum sample requirements, reduced reagent usage, shortened sample processing time, and without extensive equipment and expert technicians as needed for the traditional ELISA technique [28].

#### 3.1.3. Other Detection Techniques

SARS-CoV-2 specific antibody detection techniques, other than fluorescence, spectrometer, and image analysis-based detection platforms, are also being applied that use different detection methods to detect and quantify anti-SARS-CoV-2 antibodies in serum or plasma samples. Among them, the space encoding microfluidic biochip developed by Wang et al. can detect and quantify anti-nucleocapsid or anti-spike IgG or IgM in 60 samples simultaneously in the same run. The serum antibody can be measured by analyzing the luminescent intensity intensified with increased antibody concentrations by a GenePix 4400A microarray scanner with a limit of detection of 0.3 pg/mL, validated by detecting anti-S/N IgG and IgM in 60 serum samples. This technique requires less than 10 min for the qualitative detection of antibodies and 40 min for quantitative analysis (Table 1) [31].

Xu et al. developed an all-fiber Fresnel microfluidic biosensor (FRMB) to detect anti-S IgG or IgM in serum samples. They used a photodiode detector (PD-1000) that can quantify antibody concentrations by measuring the intensity of the Fresnel reflection light that changes along with the increase or decrease in the refractive index (RI) of the biosensing surface due to the formation of antigen-antibody complexes. This device can detect anti-S IgG/IgM within 7 min with a limit of detection of 0.82 ng/mL for IgG and 0.45 ng/mL for IgM, which is validated by testing six negative serum samples (Table 1) [32].

To determine the affinity of the antibodies towards the antigens or how strongly the antibodies bind to the antigens, as well as the concentrations of the neutralizing antibodies, Schneider et al. used a microfluidic antibody affinity profiling platform (MAAP) that applied a Biacore T200 surface plasmon resonance (SPR) system for quantifying the concentrations of nAbs against RBD and validated the results by testing 42 plasma samples from seropositive individuals (Table 1) [33].

Ko et al. developed a technique to separate the capture agent from detection for enhanced sensitivity and reproducibility and to detect anti-S-IgG against the S-RBD. PalmSens4 potentiostat is used in this technique to detect antibodies with a limit of detection of approximately 7 × 10^−12^ molecules of tetramethylbenzidine (Table 1) [34].

### 3.2. Microfluidics in Detection of SARS-CoV-2 Antigen

#### 3.2.1. Electrochemical Based Detection

Although PCR-based nucleic acid testing (NAT) is the commonly used technique for SARS-CoV-2 detection, false-negative results have also been reported with an approximate accuracy of 30–50% for confirmed COVID-19 cases. In some cases, it may be due to the sampling technique employed rather than being inherent to the test [35,36,37]. Moreover, these tests are complicated, laborious, and require a long time [38]. On the other hand, antigen kits cannot detect low viral loads [39]. The area of microfluidics provides a solution to time-consuming bench assays. Microelectronics and micro-electromechanical systems (MEMS) technologies have enabled the development of microfluidic devices capable of manipulating small amounts of fluids and extracting information from them rapidly (Table 2) [40,41].

A microelectrode array (MEA) chip that contains a specific aptamer can detect a small amount of SARS-CoV-2 N protein from the sample providing a competitive solution for real-time and low-cost SARS-CoV-2 screening and diagnosis. As an indicator, the aptasensor uses solid−liquid interface capacitance as an ultrasensitive indicator [42]. Another electrochemically-based biosensor is the MXene-graphene field-effect transistor (FET) which uses two-dimensional (2D) virus-sensing transduction material (VSTM) such as 2D transition metal carbides (MXenes) and graphene. With lower sheet resistance and superior surface chemical sensitivity than thin metal films, this is used for sensing SARS-CoV-2 spike protein. A microfluidic channel is integrated with the MXene-graphene VSTM [43].

The aptasensor has a very low limit of detection (LOD); indifferent matrices femtogram per milliliter level, the MXene graphene FET sensor can detect the antigen in a concentration ranging from 1 fg/mL to 10 pg/mL. The aptasensor has a rapid response time of 15 s and a 10−5 to 10−2 ng/mL wide linear range, although the MXene graphene FET sensor requires ∼50 min to receive viruses in solution which is much higher [42,43].

The performance of this aptasensor is validated using various types of environmental and body fluid matrices. The antigen can be detected from saliva samples and body fluids collected by routine sampling methods without culture or amplification. This aptasensor can also screen individuals at the pre-symptomatic stage or those who may be asymptomatic carriers despite costing below 1 US dollar for its ability to detect ultra-trace N-protein. This low-cost chip can be used for large-scale screening and diagnosis of SARS-CoV-2. It enables real-time detection and is easy to operate, label-free, and specific [42].

The ultra-sensitivity of the MXene−graphene FET sensor was revealed from the high signal-to-viral load ratio (~10% change in source-drain current and gate voltage), and specificity was determined using SARS-CoV-2 antibodies, producing 10 times lower signal differences. It is a relatively simple to construct, fast-responding, ultrasensitive, and specific sensor. When there is a low viral load, environmental virus sensing is used, and wearable sensing is used when direct sample collection is impossible [43].

#### 3.2.2. Fluorescence-Based Detection for Antigen Diagnosis

It was found that about 10–30% of COVID-19 patients are asymptomatic carriers, and also, patients start spreading the virus before the onset of symptoms [44,45]. Therefore, SARS-CoV-2 needs to be detected even within low concentrations. SARS-CoV-2-specific antibody detection is frequently used in conjunction with NATs. Due to the multitude of types of antibodies that appear following infection with the virus within seven days of symptom onset, the accuracy of antibody-dependent diagnosis is poor (around 40%) [46]. Protein antigen detection techniques have received much interest due to their simple detection methods to realize a non-sequencing prognosis [37,47,48].

In the digital assay, which is usually based on compartmentalized microfluidic techniques, the sample is divided into several containers containing a specific number of biological molecules. The digital enzyme-linked immunosorbent assay (dELISA) or digital PCR provides a higher degree of precision, resulting in a sensitive estimation of nucleic acids and proteins while allowing for the investigation of single-cell genotype and phenotype. Qualitative measurements can provide complete quantitative information [49,50]. Magnetic beads can be labeled with specific antibodies for dELISA, which is 100–1000 times more accurate than conventional ELISA [51,52].

A reusable microfluidic chip with femtoliter-sized wells was developed for the sensitive digital detection of N protein from SARS-CoV-2 (Table 2). On magnetic beads (MBs), β-galactosidase (β-Gal)-linked antibody specific to N protein is attached. If the sample contains N protein, it binds to the antibody on the MBs. Two aptamers (Apt 58 and Apt 61) were added, forming an immunocomplex. Following that, the MBs and β-Gal substrate fluorescein-di-β-D-galactopyranoside (FDG) were both injected into the chip. Because of the diameter of the wells, each well of the chip could only hold one MB. Fluorescent (FL) product is produced by the reaction of β-Gal and FDG in the individual femtoliter-sized wells, resulting in a locally high concentration of the FL product and sealed with fluorocarbon oil. A conventional inverted FL microscope can obtain FL images of the wells and then count the number of wells that contain MBs and the number of FL wells with MBs. The FL well percentage is determined by dividing (FL wells number) by (MBs wells number). A higher percentage of FL well corresponds to a higher percentage of N protein. The assay has a very low detection limit of 33.28 pg/mL, significantly lower than traditional sandwich ELISA. SARS-CoV-2 S protein was used as a control for assessing the kit’s specificity, which proved its excellent specificity. However, the assay has some limitations in detecting rare molecules as low bead loading efficiency. The assay’s sensitivity depends on the affinity of aptamer and antibody; higher affinity results in lower sensitivity (Table 2) [53].

To face the demands of the large epidemic, Lin et al. developed a point-of-care microfluidic platform that includes SARS-CoV-2 diagnostic microchips, a homemade fluorescence detection analyzer, and multiple immunoassays for detecting SARS-CoV-2 IgM, IgG, and antigen. The polycarbonate microchip contains a SARS-CoV-2 antigen-specific capture antibody labeled with fluorescent microsphere (FMS), which binds to the antigen present in the sample. Following that, the SARS-CoV-2 specific detection antibody binds to the complex, being immobilized by a second “antigen−antibody” affinity interaction with the SARS-CoV-2 antigen or detection antibody in the test region. The immunoassay chips are loaded into the portable fluorescence analyzer, which reads and displays the fluorescence detection results (Table 2) [54]. Unlike an inverted FL microscope, the fluorescence analyzer used here is portable, and the whole assay requires less than 15 min. The test costs only 0.71 dollars and is suitable for point-of-care testing [53,54].

In response to the pandemic, Wang et al. created a space-encoding microfluidic biochip that is fast, sensitive, and has a high throughput. The kit can be used for quantitative detection of SARS-CoV-2 antigen proteins in serum. Similar to the microfluidic device developed by Lin et al., this microfluidic biochip uses the SARS-CoV-2 specific capture antibody for binding with the SARS-CoV-2 antigen present in the serum sample. Fluorescence conjugated detection antibody then binds to the complex. GenePix 4400 was used to collect the fluorescence signals, then analyzed using GenePix 4400 software. The biochip incorporates the benefits of graphene oxide quantum dots (GOQDs) and a microfluidic chip. SARS-CoV-2 antigens and IgG/IgM antibodies from 60 serum samples were subjected to the test kit at a sample volume of 2 µL. The detection was carried out using GenePix 4400 with its corresponding software. This resulted in a detection limit for S-antigen at 4 ng/mL while the detection time was only 5 min [31].

#### 3.2.3. Spectrometer Based Detection

Several researchers have indicated that some viral antigens (i.e., S1 protein or N protein) may be found in the blood of people who experience coronavirus viremia [55,56]. Tan et al. created a portable microfluidic ELISA technology to detect SARS-CoV-2 S1 and N antigens within 40 min using a spiked serum. The capillary sensor array is polystyrene with 12 channels in this microfluidic chemiluminescent ELISA system. The authors used the conventional sandwich ELISA and the poly-HRP signal amplification technique to detect viral antigens with high sensitivity. SARS-CoV-2 S1 RBD-specific antibody and SARS-CoV-2 S1-specific antibody were used as the capture and detection antibody, respectively. In contrast, for the detection of N protein, SARS-CoV-2 N-specific antibody and SARS-CoV-2 N-specific antibody were used as the capture and detection antibody, respectively. The biotinylation of detection antibodies and the incorporation of poly-HRP led to a 5–10 times enhancement of the detection limit, thereby improving the sensitivity of the assay. The lower limit of detection (LLOD) for S1 was 4 pg/mL, whereas it was 62 pg/mL for N using a UV-Vis spectrometer [30].

#### 3.2.4. Other Detection Techniques

The current COVID-19 serological test uses lateral flow assay (LFA) or ELISA for the identification of anti-SARS-CoV-2 IgG and IgM [57,58,59,60]. Based on these assays, it has been observed that virus-specific antibodies appear 2–3 weeks after infection. Henceforth, these assays are unable to detect early-stage infection in comparison to antigen as well as molecular assay [61,62].

SARS-CoV-2 spike (S1) and N proteins have been reported to be found at a range above 8 pg/mL and 0.8 pg/mL, respectively [63]. Another study claims 92 and 97 percent sensitivity and specificity, respectively, in the detection of SARS-CoV-2 nucleocapsid (N) protein COVID-19 patient serum [62]. These immunoassays require a long time, approximately 3–4 h, as they include liquid handling steps and are laborious despite being commercially available [64]. Immunosensors were developed by several research groups for early screening of SARS-CoV-2 antigens. The microfluidic chemiluminescent ELISA system, created by Tan et al., can detect SARS-CoV-2 S1 and N proteins within 40 min at a 10-fold dilution of patient serum [30]. Another multiplexed electrochemical immunoassay, described by Torrente-Rodrguez et al., could detect SARS-CoV-2 N protein as well as SARS-CoV-2 S1 antibodies [65]. Fabiani et al. also described a similar immunosensor that can detect SARS-CoV-2 S1 and N proteins in saliva at the lowest concentration, of 19 ng/mL and 8 ng/mL, respectively (Table 2) [66].

Though these immunoassays can identify SARS-CoV-2 antigens in biofluid specimens, they either require high sample dilution or lack high sensitivity (pg/mL). An immunosensor employing dually-labeled magnetic nanobeads is able to detect SARS-CoV-2 N protein from undiluted serum at pg/mL levels within an hour while requiring only a 25 µL sample and 80 µL of reagent. This is possible due to the incorporation of immunomagnetic enrichment as well as signal amplification by the labeled magnetic nanobeads. The main components of the assay are the neodymium magnet labeled with HRP and detection antibody (dAb) and screen-printed gold electrode (SPGE) sensors with N protein capture antibody. The reduction of TMB catalyzed by HRP will result in a current potential that is proportional to the concentration of the target antigen on the sensor surface. SARS-CoV-2 N protein concentrations in spiked samples were detected at 10 pg/mL and 50 pg/mL in diluted serum and whole serum, respectively. The further incorporation of immunosensors into smartphones allows for high sensitivity and specificity. Clinical testing of the device differentiated PCR-positive COVID-19 patients from healthy, uninfected individuals presents a promising tool for point-of-care COVID-19 testing (Table 2) [64].

In this pandemic scenario, masking along with social distancing remains the best prevention strategy for COVID-19 prevention [67]. Many people’s unwillingness to wear masks calls for the need to detect SARS-CoV-2 in the air [68,69]. An innovative microfluidic system uses a simple sprayer in a chamber simulating a human cough to collect droplets/aerosols inactively on a paper microfluidic chip. Later antibody-antigen binding and particle aggression by introducing antibody-conjugated submicron fluorescent particles on the chip. The chip is made of nitrocellulose paper containing four channels. A low-cost smartphone-based fluorescence microscope was built, and the microscopic images were used to quantify the extent of particle aggregation and confirm the presence of SARS-CoV-2 in the air. With a size of 10 cm × 5 cm × 10 cm, the device is adjustable and portable and can be configured to detect other respiratory viruses and bacteria just by changing the antibody and optimizing the particle concentration (Table 2) [70].

By merging ultrahigh throughput hydrodynamic filtration and sandwich immunoassay, a novel microfluidic test kit has been developed for the naked-eye detection of SARS-CoV-2. Compared with traditional LFA and microfluidic platforms, which have antigen binding and separation on the same substrate, this handheld microfluidic test kit’s incubation (antigen-antibody binding) and signal detection (separation of unbound antigens) are carried out within a test tube and a microfluidic filtration chip, respectively. Moreover, the kit produces immediate, naked-eye visible results without fluorescence or an optoelectronic detector. To retain the N proteins of the SARS-CoV-2, nano and microbeads were coated with two different, non-competitive antibodies simultaneously, forming larger complexes. Free nanobeads are discarded during microfluidic filtration, but antigen-bridged complexes are retained in the observation zone, where a red display indicates the presence of antigen in the sample. With an LLoD of 100 copies mL^−1^, this testing prototype has a high throughput separation (the 30 s) and antigen enrichment that outperforms conventional lateral flow or microfluidic assays. With high sensitivity (overall 95.4%) and specificity (100%), this microfluidic test kit can detect SARS-CoV-2 virus variants developed over significant periods. It is durable and straightforward and can be used for point-of-care or self-service testing. Furthermore, the chip can be reused more than 50 times, and the mass-produced chip can be produced for $0.98 per test. Because of these features, the microfluidic test kit is ideal for resource-poor settings (Table 2) [71].

Paper-based microfluidic platforms are being increasingly incorporated into research, environmental monitoring, medical diagnosis, as well as biochemical analysis, owing to them being inexpensive and requiring less preparation with almost no requirement for complex peripheral equipment [72]. Mingdi Sun et al. developed a paper-based microfluidic platform using Whatman 3 MM Filtre of 50 mm diameter. It had eight reaction areas, each of 5 mm diameter with a sampling area of similar diameter and a waste liquid area encircling it. A coating antibody was cross-linked with the chitosan-glutaraldehyde method, while the SARS-CoV-2 N antigen detection was carried out by the sandwich ELISA method. The system was able to detect N antigen at concentrations as low as 8 μg/mL with high sensitivity, specificity, and reproducibility [73].

Jadhav et al. developed Au/Ag coated carbon nanotubes conjugated with surface-enhanced Raman spectroscopy (SERS), which successfully identified viruses from multiple biological specimens. The device implemented size-dependent trapping of SARS-CoV-2 while being identified by their Raman signature [74,75,76]. The device was cost-effective as well as less time-consuming. Based on the implementation of micro/nanofilters or vertically aligned carbon nanotubes (VACNTs) on a PDMS, the device can be configured into two models (Table 2) [74].

Among the microfluidic devices, the LumiraDx SARS-CoV-2 Ag test received EUA as early as August 2020. The device is able to detect SARS-CoV-2 nucleocapsid antigens from patient nasal swabs [77]. In a study, the device identified 95.8% of samples in less than 33 RT-PCR cycles and 100% in less than 30 RT-PCR cycles [78]. The results were reproduced in another study involving 1232 patients, with a 97% concordance with Nucleic Acid Amplification Tests (NAAT)+ samples, which were identified using multiple EUA-approved NAAT kits (Table 2) [79].

### 3.3. Detection of SARS-CoV-2 Nucleic Acids through Microfluidics

#### 3.3.1. Fluorescence-Based Detection of Nucleic Acids

##### RT-qPCR Based Amplification

Fluorescence is the most extensively used approach due to its high sensitivity and abundance of fluorophores and labeling chemistries [80]. A quantitative nanofluidic assay based on qPCR developed by Fassy et al., three-step microfluidic nano-scale qPCR based on a microfluidic chip designed by Xie et al., microfluidic chip PCR technology developed by Francesca Dragoni et al., microfluidic disc-direct RT-qPCR assay developed by Ji M et al., portable MiDAS for SARS-CoV-2 nucleic acids detection developed by Yang, J et al., detect SARS-CoV-2 nucleic acids by the fluorescence-based detector (Table 3) [81,82,83,84,85].

**Table 2:** Microfluidic SARS-CoV-2 antigen detection kit.

Study ID	Methods	Fluid Manipulation Technique	Material	Immobilized Antigen/Antibody/Gene	Detected Biomolecules	Detector	Sensitivity	Specificity	Sample Size/Donor/Standard	Limit of Detection (LOD)	Detection Time	Advantages
Lin 2021 [22]	Fluorescence immunoassay	Centrifugal force	Polycarbonate	FMS coated SARS-CoV-2 capture antibody	SAS-CoV-2 antigen	homemade fluorescence detection analyzer	NR	NR	10 patient and 9 healthy people	NR	˂15 min	Portable, rapid, easy to use, on-site detection, high sensitivity, and specificity.
Tan 2020 [30]	Microfluidic chemiluminescent ELISA technique	Capillary force	Polystyrene	Capture antibodies to S1 and N	N and S	NanoDrop™ UV-Vis spectrophotometer	NR	NR	16 convalescent patients and 3 healthy participants	4 pg/mL (S) and 62 pg/mL (N)	40 min	Low time consumption, sensitive, low sample volume requirement, low-detection limit
Wang 2021 [31]	Space-encoding microfluidic biochip	Pump	PDMS	Capture antibodies to S and N	N and S	GenePix 4400A Microarray Scanner	NR	NR	60 serum samples	~0.3 pg/mL to ~4 ng/mL	˂10 min (qualitative) 40 min (quantitative)	60 sample per test, fast, sensitive, Ultralow detection limit
Qi H, 2022 [42]	MEA chip based solid−liquid interface capacitance/ trace N-protein detection by microfluidics-coupled capacitive sensor	DEP force (Pneumatic micropumps)	MEA chip modified with an aptamer	An aptamer for SARS-CoV-2 N protein	SARS-CoV-2 N	Sensor and impedance analyzer	NR	NR	0.1 mL saliva sample collected from 3 volunteer	1.26 × 10^−6^ ng/mL (saliva) 2.16 × 10^−6^ (plasma) and 1.82 × 10^−6^ ng/mL, (serum)	15 s	Wide linear range from 10^−5^ to 10^−2^ ng/mL, a real-time, easy-to-operate, label-free, and specific
Li Y, 2021 [43]	MXene–graphene field-effect transistor (FET) sensor to create an ultra-sensitive VSTM	NR	PDMS	APTES linked Anti-S IgG	SARS-CoV-2 spike protein purchased from SinoBiological	fabricated MXene− graphene FET sensor	NR	NR	recombinant 2019-nCoV spike protein	1 fg/mL	∼50 min	Relatively simple to construct, fast-responding, ultrasensitive, and specific sensor
Ge C, 2022 [53]	Microfluidic chip with femtoliter-sized wells, Biotinylated aptamer and β-Galactosidase affinity	Peristaltic pump	PDMS	Capture-SA-β-Gal-linked anti-N IgG Detection- Biotinylated aptamers	SARS-CoV-2 N	Inverted fluorescent microscope	NR	NR	SARS-CoV-2 N (Suzhou) Biotecnology Co., Ltd.)	33.28 pg/mL	NR	Simple, cost effective, detection by fluorescence, reusable
Li J, 2021[64]	Microfluidic chip with an integrated immunosensor that utilizes dually labeled magnetic nanobeads	Electromagnetic micropump	PET film stacked with a PMMA cartridge on top of an SPGE sensor	Dually labelled magnetic nanobeads with HRP and detection antibody	SARS-CoV-2 N	Microfluidic immunosensor chip	NR	NR	SARS-CoV-2 N (Advaite, Inc.)	100 pg/mL (5× diluted serum) and 230 pg/mL (whole serum)	<1 h	Portable, simple, and highly sensitive immunosensor
S Kim, 2021 [70]	Airborne droplets are captured on the paper microfluidic chip and detected by fluorescent conjugated antibody	Capillary manipulation	Nitrocellulose paper	Detection antibody conjugated with yellow-green fluorescent carboxylated polystyrene particles	SARS-CoV-2 N	Smartphone-based fluorescence microscopic imaging	NR	NR	SARS-CoV-2 Isolate USA-WA1/2020	NR	<30 min	Low cost, handheld, foldable paper microfluidic assay, rapid virus detection from air droplets
Xu J, 2021 [71]	Hydrodynamic filtration with sandwich immunoassay	Syringe pump	PDMS	N-MAb conjugated in white microbead and red nanobead	SARS-CoV-2 N	Naked eye detection	95.4%	100%	93 individuals (48 negatives and 45 positives by qPCR)	<100 copies/ mL	NR	Simple to use, point-of-care, reusable and cost-effective chip,
Sun M, 2022 [73]	Chitosan-glutaraldehyde cross-linking to coated antibody, and sandwich ELISA for detection	Capillary manipulation	Whatman 3 MM filter paper	Capture-N Specific MAb Detection-HRP-tagged MAb	SARS-CoV-2 N	Camera and ImageJ software	NR	NR	N protein (Guangzhou Qianxun Biotechnology Co., Ltd., Guangzhou, China)	8 μg/mL	NR	Small-sized chip, simple and easy portable, rapid detection

Abbreviations: FDG—fluorescein-di-β-D-galactopyranoside; β-gal—β-galactosidase; MEA—microelectrode array; VSTM—virus-sensing transduction material; FET—field-effect transistor; APTES—3-aminopropyl triethoxysilane; LLOD—lower limit of detection; PET—polyethylene terephthalate; PMMA—poly(methyl methacrylate); SPGE—Screen-printed gold electrode.

Quantitative Real-Time RT-PCR (qRT-PCR), along with high-throughput sequencing, is the most ubiquitous technique implemented for the detection of SARS-CoV-2 [86]. Although qRT-PCR provides acceptable sensitivity for early infection identification, the possibility of false-negative or false-positive findings is one of the most critical clinical factors for pandemic transmission [87]. Such findings occur when the viral content is low, as evident in asymptomatic or weakly positive individuals [88]. Henceforth, timely diagnosis is required to avoid such outcomes [89]. To address the issue of false-negative results while simultaneously being able to detect low viral loads that are missed in the RT-PCR technique, Xie et al. developed a three-step microfluidic qPCR method that incorporates reverse transcription, targeted cDNA pre-amplification, which enables detection below 1 copy/µL and nanoscale PCR methods [82]. Fassy et al. also included the pre-amplification step enabling the detection of seven transcript copies per reaction for N genes. 

The qPCR-based nanofluidic assays can perform 4608 qPCR of 12 pairs of primer within three hours or less. This translates to 192 clinical samples at one time, reducing reagent consumption and providing flexibility in probe inclusion [81]. Ji M et al. developed a microfluidic disc-direct RT-qPCR (dirt-qPCR) assay that is able to detect viral infection by simultaneously detecting up to 16 targets. The procedure takes 1.5 h with an LOD of 2 × 10^1^ copies/reaction. In comparison with traditional RT-qPCR, the technology had a consistency rate of 99.54, 99.25, and 100% for Influenza A, Influenza B, and SARS-CoV-2 (Table 3) [84].

Microfluidic chip-based PCR technology such as SHINEWAY SWM-01 Nucleic Acids Analyzer (SWM-01) is able to simultaneously detect viral components from three to nine samples on a single microchip with three channels within 45 min by incorporating single-channel fluorescence with microfluidic chip technology. On the other hand, weakly positive samples are wrongly allocated to this new analyzer, potentially due to the instrument’s detection limit, which is a significant disadvantage over other techniques [83]. A photomultiplier tube (PMT) light detector collects fluorescence in the dirRT-qPCR assay. On the other hand, the fluorescence signal is collected as images from a CMOS camera using microfluidic chip-based PCR technology [83,84]. A portable microfluidic-based integrated detection analysis system (MiDAS) developed by Yang, J et al. detects SARS-CoV-2 nucleic acids in saliva samples in less than two hours with a 1000 copies/mL limit of detection [85]. For COVID-19 molecular testing, NP swabs are considered a standard sample source. However, because NP swab collection requires skilled individuals, it limits consumer accessibility. In a systematic meta-analysis that explored the sensitivity of saliva and nasopharyngeal swab nucleic acid amplification testing, the authors found a sensitivity of 83.2% and 84.8%, respectively, and a specificity of 99.2% and 98.9%, respectively [90]. However, other studies present saliva as a good alternative biofluid sample [91,92,93]. The MiDAS SARS-CoV-2 system, employing detection of antigens from saliva, can be deployed in areas/countries with limited resources due to the low cost, lack of cross-contamination, automation, as well as high sensitivity followed mobility [85]. The detected biomolecules are cDNA for all of the stated assays (Table 3).

Kim HS et al. developed a microfluidic device incorporating rolling circle amplification in a mesh with multiple pores which can detect DNA. Using DNA concentrations of 3.0 or 30 aM, SARS-CoV-2 can be detected within 5 or 15 min. The decreased testing time is attributed to the micro-scale holes generated in the mesh, which can readily be blocked by RCA gelation. The kit also presents the potential to be implemented in any location and requires no power source(Table 3) [94].

##### RT-LAMP Based Amplification

Isothermal nucleic acid amplification eliminates the requirement of thermocycling, making NAAT more quick and convenient than polymerase chain reaction in identifying infections [95,96,97,98,99]. The need for nucleic acid extraction and amplification in expensive instrumentation, as well as the requirement of high-level biosafety, laboratories have hampered the implementation of NAAT for disease diagnosis outside of a clinical laboratory setup [100,101]. Tian et al. developed an automated centrifugal microfluidic system with reverse-transcriptase loop-mediated isothermal amplification (RT-LAMP)-based amplification for SARS-CoV-2 RNA detection eliminating the use of expert operators or diagnostic facilities while at the same time improving its detection capabilities. The whole process following injection of oropharyngeal swab sample into the microfluidic disc, such as sample treatment, RT-LAMP, and signal detection, was automated. The whole assay was completed within 70 min with an LOD of two copies/reaction [102]. Xiong H et al. developed a rotating microfluidic fluorescence system detection based on RT-LAMP, Ramachandran A et al. developed isotachophoresis coupled RT-LAMP based amplification and clustered regularly interspaced short palindromic repeats (CRISPR)–Cas12 based detection, Huang Q et al. developed a portable microfluidic chip-based system with two-stage isothermal amplification, Soares et al. developed the modular centrifugal microfluidic platform to perform RT-LAMP, all detect nucleic acids of SARS-CoV-2 by fluorescence-based detection [103,104,105,106]. The limit of detection of rotating microfluidic fluorescence systems detection based on RT-LAMP and isotachophoresis coupled RT-LAMP based amplification and CRISPR–Cas12 based detection is the same (10 copies/μL). However, the rotating microfluidic fluorescence system shows 100% specificity, 91.82% sensitivity, and precision for rapid detection of SARS-CoV-2 within 15 min (Table 3) [103,104]. Ganguli et al. also developed a portable microfluidic device capable of detecting SARS-CoV-2 within 40 min using smartphone-based fluorescence imaging. With a sample size of 20, the device presented high reproducibility along with 100% accuracy, sensitivity, and specificity. Additionally, the device also demonstrated a LOD of 50 copies/μL, which can be claimed to be significant. Another advantage of the device is that it does not require RNA extraction, thereby facilitating its implementation as a point-of-care device [97].

The rotating microfluidic fluorescence system incorporates the use of a small sample volume by incorporating automated centrifugal force as well as an air rotation heating system into the sample injection system, enabling rapid nucleic acid amplification for early screening of SARS-CoV-2 nucleic acid (Table 3) [103]. On the contrary, Ramachandran A et al. employed isotachophoresis, an electrokinetic microfluidic technique (ITP). For CRISPR reactions, the microfluidic method uses a small amount of reagents on-chip (order 100-fold less than conventional methods) and can be automated, allowing detection within 30 to 40 min [104]. Another bead-based signal enhanced, centrifugal microfluidic platform detected SARS-CoV-2 within 1 h, for 162 NP samples with Ct values below 26, with a specificity of 100% and a sensitivity of 96.6% using a smartphone read-out (Table 3) [106]. A microfluidic-chip-based system can carry out parallel detection of multiple targets (22 targets, both DNA RNA) with a two-stage isothermal amplification method; recombinase polymerase amplification (RPA) in the first stage and fluorescence LAMP in the second stage within an hour without any cross-contamination and it is an excellent advantage over the other stated techniques. This assay demonstrated 94.12% specificity and 95.83% sensitivity for SARS-CoV-2 with a very low detection limit of about 10 copies. So, it is ideal for resource-constrained areas and point-of-care testing (POCT) (Table 3) [105].

#### 3.3.2. Other Nucleic Acid Detection Techniques

Yang et al. developed ultrasensitive isothermal amplification and a microfluidic POC (point of care) diagnosis system based on the PTS (MPSP). Li et al. developed CRISPR-based recognition SARS-CoV-2 amplified gene by RPA in a microfluidic chamber and AuNP conjugated lateral-flow system for detection. The naked eye can detect SARS-CoV-2 nucleic acids [107,108]. The whole detection process of SARS-CoV-2 in Yang et al.’s developed assay took less than 2 h with a limit of detection of 0.5 copy/µL and was validated by diagnosing one clinical authenticated swab sample from a COVID-19-positive patient and 16 negative samples [107]. On the contrary, the detection limit in Li et al. developed assay is 100 copies of RNA per target. The outcomes of this CRISPR-based microfluidic system were validated by successfully detecting SARS-CoV-2 in 24 clinical nasopharyngeal swab samples with sensitivity (94.1%), specificity (100%), and accuracy (95.8%) [108]. Yin et al. developed a three-dimensional microfluidic chip with a two-stage amplification with the first incorporating recombinase polymerase amplification (RPA) and the second incorporating synergetic enhanced colorimetric, loop-mediated isothermal amplification (SEC-LAMP). The colorimetric signal is read by the naked eye or a smartphone with a high sensitivity of 10 genome equivalent/mL within an hour (Table 3) [109].

By combining multiple responsive molecular nanostructures forming catalytic molecular circuitry and automating microfluidics, Zhao et al. were able to develop an integrated platform for accurate as well as easy detection of SARS-CoV-2 nucleic acids. Pressure actuation and liquid guiders are used in automated microfluidics to coordinate the numerous molecular processes, resulting in a smooth conversion of the target-induced molecular activation into an increased electrochemical signal recorded using a miniaturized potentiostat (PalmSens, EmStat3) during the entire detection process. This method catalyzes signal intensification from target hybridization instead of depending on target amplification as traditional nucleic acid detection. Even against the complicated biological backdrop of original clinical samples, direct RNA measurement is possible that avoids all processing stages of standard RT-qPCR. The eSIREN platform detects SARS-CoV-2 in less than 20 min with a sensitivity of 92.3%, specificity of 87.5%, and overall accuracy of 90.5% among tested 21 clinical samples (Table 3) [110].

**Table 3:** Microfluidic nucleic acid detection kit for SARS-CoV-2.

Study ID	Methods	Fluid Manipulation Technique	Material	Immobilized Antigen/Antibody/Gene	Detected Biomolecules	Detector	Sensitivity	Specificity	Sample Size/Donor/Standard	Limit of Detection (LOD)	Detection Time	Advantages
Fassy 2021 [81]	Quantitative nanofluidic assay based on qPCR	Manual pipetting	192.24 IFC	N, E, ORF1ab, S, NSP6 gene and mutants	cDNA	Fluorescence based detection	NR	NR	20 clinical samples	7 transcript copies per reaction (for N gene)	<3 h for 192 samples	192 samples in single run, multiple targets
Xie 2020 [82]	3 step microfluidic nano-scale qPCR based on microfluidic chip	Manual pipetting	192.24 IFC	N gene	cDNA	Fluorescence based detection	NR	NR	182 NP swab samples from 91 positive and 91 negative participants	˂1 copy/µL	NR	Increased throughput, high precision
Francesca Dragoni, 2021 [83]	Microfluidic chip PCR technology	NR	NR	RT-qPCR of ORF1ab and N gene	cDNA	Fluorescence based detection	NR	NR	20 samples	Ct < 36	45 min	Easy, Fast, Quantification of viral RNA is possible, Small amount of reagents needed.
Ji M, 2020 [84]	Microfluidic disc-direct RT-qPCR assay	Centrifugal force	PMMA	N-gene	cDNA	Fluorescence based detection	NR	NR	29 SARS-CoV-2, and 1572 negative samples	2 × 10^1^ copies/reaction	1.5 h	Fast, High sensitivity, Automation capability, Direct viral detection from sample
Yang, J; 2021 [85]	Portable MiDAS for SARS-CoV-2 nucleic acids detection	Electrochemical pumping	Polycarbonate	1-Step RT-qPCR based amplification of N gene	cDNA	Fluorescence based detection	NR	NR	200μL saliva spiked with SARS-CoV2 RNA and/or γ-irradiation inactivated SARS-CoV-2 virions	1000 copies/mL	˂2 h	Rapid, Sensitive, Cheap, Automation capability, Cross-contamination is avoided.
Kim HS, 2021 [94]	RCA of pathogen specific gene amplification on a mesh having multiple microfluidic pores	Hydrostatic pressure	Nylon	RCA based amplification of SARS-CoV-2 nucleic acids.	DNA	Fluorescence based detection	NR	NR	Nucleic acid sequences (20 nt) for COVID-19 (synthesized by Genotech Daejeon, Korea)	0.7 aM	≤5 or 15 min	Easy, Effective, Rapid, Does not require any sophisticated device, simple operating principle, Can operate without accessible electricity.
Ganguli 2020 [97]	Microfluidic system based on RT-LAMP	NR	NR	ORF1a, ORF8, S and N gene	RNA	Fluorescence based detection	100%	100%	20 clinical samles	50 copies/μL	40 min	Does not reuire RNA extraction
Tian F, 2020 [102]	Automated centrifugal microfluidic system with RT-LAMP-based amplification	Centrifugal force	PMMA	N gene specific RT-LAMP primers	cDNA	Fluorescence based detection	NR	NR	Plasmids containing the N gene	2 copies/reaction	≤70 min	Rapid, Sensitive, Specific, Viral contamination of aerosol is avoided
Xiong H, 2021 [103]	Rotating microfluidic fluorescence System, detection based on RT-LAMP	Centrifugal force	Polycarbonate	ORF1ab and N gene	cDNA	Fluorescence based detection	91.82%	100%	115	10 copies/μL	15 min	Rapid, portable, Highly sensitive, Well precision
Ramachandran A, 2020 [104]	Isotachophoresis coupled RT-LAMP based amplification and CRISPR–Cas12 based detection.	Isotachophoresis	Glass	E and N gene	cDNA	Fluorescence based detection	NR	NR	Synthetic ssRNA	10 copies/μL	35 min	Minimal reagent consumption, rapid detection, simple sample processing
Huang Q, 2021 [105]	Microfluidic-chip-based system with two-stage isothermal amplification method; RPA in the first stage and fluorescence LAMP in the second stage	Capillary action	PMMA	S gene	cDNA	Fluorescence based detection	95.83%	94.12%	Plasmid DNA, 17 clinical nasopharyngeal swab	10 copies	Around 1 h	Parallel detection of multiple target accurately, Rapid detection with high specificity and sensitivity
Soares, 2021 [106]	Modular centrifugal microfluidic platform to perform RT-LAMP	Centrifugal force	PMMA, PDMS	ORF1ab gene	cDNA	Fluorescence based detection	96.6%	100%	162 nasopharyngeal swab	100 RNA copies in 10 μL	1 h	Scalable, rapid, and sensitive
Yang 2021 [107]	Ultrasensitive isothermal amplification along with microfluidic POC diagnosis system based on the PTS (MPSP)	Manual pipetting followed by capillary action	NR	M and N genes	cDNA	Naked eye detection	NR	NR	1 clinical authenticated swab sample from COVID-19 positive patient and 16 negative samples of different viruses	0.5 copy/μL	<2 h	High-throughput, on-site detection of multiple viruses
Li 2021 [108]	CRISPR-based recognition of SARS-CoV-2 amplified gene by RPA in a microfluidic chamber and AuNP conjugated lateral-flow system for detection.	Capillary action	Clear resin	N-gene	In-direct detection of cDNA	Naked eye detection	94.1%	100%	24 clinical nasopharyngeal sample	100 copies RNA/target	NR	Easy to use, portable, low cost, no requirement of electricity, high sensitivity, specificity and accuracy, contamination free.
Yin 2021 [109]	SMCD based integrated on-chip nucleic acid extraction, two-stage isothermal amplification (RPA and LAMP), and colorimetric detection on a 3D printed microfluidic chip	Syringe pump	Clear methacrylate-based resin	N gene, E gene, and Orf1a gene	cDNA	Naked eye detection	100 GE/mL	NR	7 samples	NR	≤1 h	Portable on site detection, low cost, convenient, rapid detection, higher sensitivity and specificity, smartphone-based visualization
Zhao H, 2021 [110]	eSIREN	Electrochemical pumping	PDMS, PMMA	In-direct detection of SARS-CoV-2 S-gene	RNA	Miniaturized potentiostat (PalmSens, EmStat3)	92.3%	87.5%	21 samples	7 copies of target RNA/μL	<20 min	Accurate detectio, Reaction operates at room temperature, in-direct viral RNA detection

Abbreviations: MiDAS—microfluidic-based integrated detection analysis system; eSIREN—electrochemical system integrating reconfigurable enzyme-DNA nanostructures; RT-LAMP—reverese transcriptase-loop-mediated isothermal amplification; gRNA—guide RNA; RPA—recombinase polymerase amplification; SMCD—Sensitive multiplexed colorimetric detection; FTA—Flinders Technology Associate; PTSs—Portable commercial pregnancy test strips; GE—genome equivalent; AuNP—Gold nanoparticle; RCA—rolling circle amplification; PMMA—Polymethyl methacrylate; PDMS—Polydimethylsiloxane.

## 4. Material and Fluid Manipulation Technique

Different materials are used for fabricating microfluidic systems, such as silicon, glass, paper, and polymer (Table 1, Table 2 and Table 3) [80,111,112]. Poly(methyl methacrylate) (PMMA), polystyrene, polycarbonate, and PDMS are examples of polymer materials. PDMS has become one of the most widely utilized polymer materials for microfluidic devices in recent years because it is flexible, optically transparent, and biocompatible [80].

The selected studies used several materials and fluid manipulation techniques in this review. As for the material, PMMA was frequently used, followed by PDMS, polycarbonate, 192.24 IFC, nylon, glass, clear resin, and clear methacrylate-based resin [81,84,85,94,102,104,108,109,110]. Among fluid manipulation techniques, centrifugal force was the most commonly used strategy, followed by electrochemical pumping, capillary action, manual pipetting, hydrostatic pressure, isotachophoresis, and syringe pump [82,84,85,94,102,104,105,109]. Yang et al. used manual pipetting followed by capillary action for the fluid manipulation strategy [107].

## 5. Microfluidic Devices beyond SARS-CoV-2 Diagnosis

The clinical spectrum of COVID-19 spans from asymptomatic to mild to moderate self-limiting disease [113,114,115]. However, in some patients, comorbidities such as cardiovascular/pulmonary disease and diabetes can result in severe and fatal consequences [116,117]. In severe COVID-19 cases, the pulmonary system is mainly involved. Furthermore, evidence suggests that SARS-CoV-2 has a wide tropism in the kidneys, heart, large intestines, spleen, and liver [118,119]. The virus’s primary cellular target is the ACE-2 [120]. As evidenced by the detection of virus-like particles in the pulmonary and kidney endothelium of COVID-19 patients, ACE2 is expressed throughout the body’s vasculature, allowing SARS-CoV-2 entry to various organ systems [121]. In terms of the central nervous system (CNS), ACE-2 is also found in the human cerebral vasculature [122,123].

An advanced 3D microfluidic model mimicking the human blood-brain barrier (BBB) was fabricated by polymerizing hydrogels composed of 5 mg/mL hyaluronan, 1 mg/mL type I collagen, and 1 mg/mL Matrigel. The device provided the physiological conditions of the CNS interface to demonstrate that s1 promotes loss of barrier integrity. The addition of spike proteins to in vitro BBB models resulted in significant changes in barrier properties. The findings presented in this report looked into whether the SARS-CoV-2 viral spike protein had any adverse effects on primary human brain microvascular endothelial cells (hBMVECs). The spike protein, which is essential for receptor recognition, comprises the S1 subunit, an RBD, and the S2 subunit. The study first demonstrated that ACE-2 is widely expressed in the frontal cortex using postmortem brain tissue, and its expression was found to be increased in cases of dementia and hypertension. According to the evidence, the SARS-CoV-2 spike proteins cause a pro-inflammatory response in brain endothelial cells, contributing to an altered BBB function state. The findings demonstrated the direct impact of SARS-CoV-2 spike protein on the brain endothelial cells providing an explanation for the neurological consequences observed in some patients after COVID-19 infection [124].

COVID-19 patients with severe illness present with a high frequency of thrombotic pathophysiologies, such as venous thromboembolism, microvascular thrombosis, and acute arterial thrombosis. The thrombosis rate in hospitalized patients approaches 20% despite standard prophylactic anticoagulant administration [125,126].

Innovative therapies for preventing thrombosis in COVID-19 patients mostly involve animal models as well as early phase human trials due to a lack of in-vitro methods for such exploration. To address this particular issue, a microfluidic chip made of PDMS with two orthogonal channels, where one simulates the blood vessels while the other represents the bleeding channel to simulate hemostatic plug formation. A set of three pillars at the intersection between the two channels, coated with collagen, is placed for platelet adhesion as well as aggregation [127].

Current COVID-19 prevention strategies are vaccination and neutralizing antibodies, which prevent the binding of S proteins to ACE-2 receptors [20,128,129]. The clinical importance of these techniques has been debated though they remain undeniably valid. Vaccine development, as well as production, remains incompatible with an emergency time frame, in addition to the fact that it is more of a preventive measure than a therapeutic one in severe COVID-19 cases [130]. As a result, alternative therapeutic measures that provide comprehensive therapeutic advantages are precious for impactful COVID-19 treatment. An inhaled microfluidic microsphere with a genetically engineered membrane from ACE-2 receptor-overexpressing cells and macrophages competes with the virus for binding with ACE-2. The inhaled microspheres significantly decrease SARS-CoV-2 pathogenic efficiency across the respiratory system while relieving the lymph node and spleen hyperinflammation and neutralizing pro-inflammatory cytokines, effectively demonstrating significant therapeutic efficacy [131].

Timely screening, careful supervision of cytokine storms as well as timely guidelines for anti-inflammatory treatments to achieve better survival rates are critical due to the dynamism of the COVID-19 evolution. Microfluidics incorporates the tests while being simple to use and cost-effective [132,133]. To meet the growing demand for COVID-19 cytokine storm monitoring, a machine-learning-assisted microfluidic nano-plasmonic digital immunoassay was developed with three notable features: high-throughput, multi antibody-arrayed biosensing chip, and microfluidic microarray patterning. The device’s ultrasensitive nano-plasmonic digital imaging technology utilizes 100 nm silver nano-cubes (AgNCs) and machine-learning-based image processing for signal transduction and digital signal analysis, respectively. The assay has a very low detection limit down to sub pg mL^−1^. It was evaluated using serum specimens from 40 severe COVID-19 patients. The assay measured six cytokines in these samples, and the results showed elevated serum cytokine levels in these patients. On further serum cytokine profiling from different patients, the immunoassay presented high sensitivity, precision, and wide dynamic range in serum cytokine assessment [134].

The “pre-equilibrium digital enzyme-linked immunosorbent assay (PEdELISA) microarray” is a highly multiplexed digital immunoassay platform. The PEdELISA microarray analysis addresses limitations associated with digital multiplexing by incorporating small footprint on-chip biosensors and fully automating the signal counting process. Its microfluidic spatial-spectral encoding method and a machine learning-based image processing algorithm increase the multiplexing capacity of the device. Color-encoded magnetic beads allow the assay reaction to be conducted on-chip (no bead loss) entirely, requiring only a 15 µL sample volume, a 5-min assay incubation time, and a chip size of 75 mm by 50 mm. The device can quantify a large panel of biomarkers at the same time without compromising quality. It also can facilitate acute immune disorder monitoring that guides timely treatment plans due to its quick assay turnaround and analytical power. After analyzing longitudinal blood samples from human patients who had cytokine release syndrome (CRS) after receiving CAR-T therapy, the data showed the evolution of 12 circulating cytokines throughout illness development [135].

Organ-on-chip (OOCs) are microfluidic devices that enable the study of drugs, development, and others by mimicking the physiological as well as biochemical characteristics of the functional units of the organ. The chips themselves are a few centimeters in size, while the microchannels are micrometers in size [136]. Such devices are prepared from polymers such as PDMS, PVDF, nitrocellulose, or polyester in accordance with the desired cell adhesion as well viability properties being studied. The implementation of such devices mimicking the organs, in lieu of animal testing, helps in studying the underlying mechanisms of SARS-CoV-2 infection and pathogenesis while being quick and low-cost [137].

A better understanding of the massive scale of viral transmission as well as the evolution of clinically significant variant strains necessitates more genomic data. These are generally generated using targeted, whole-genome amplification and next-generation sequencing (NGS) [138,139,140,141].

Traditional multiplexing RT-PCR processes require a sophisticated primer design as well as experienced hands-on skills to minimize contamination as well as operator errors. Li et al. developed an integrated microfluidic nucleic acid amplification system based on one-step RT-PCR. Incorporating an in-house developed bioinformatics pipeline following NGS amplicon sequencing results in a robust and efficient way of obtaining SARS-CoV-2 complete genome sequences [142].

## 6. Conclusions and Future Direction

Though research in microfluidics has been advancing for almost a half-century, its adoption into real-world applications has been slow and has encountered hurdles. During recent decades, with advancements in material science and microfluidic device manufacturing techniques, the technology has seen a surge in its uptake in diagnostics. Furthermore, the implementation of miniaturization of detection techniques facilitated its incorporation within devices, making its deployment much more ubiquitous. With further advances in the implementation of fluid manipulation techniques along with developments in material sciences, the implementation of miniaturized biosensors with high signal processing capacity will inadvertently allow the execution of personal diagnostics within the population will nonetheless lead to better treatment regimens for the patients.

The COVID-19 pandemic has truly unleashed different microfluidic techniques that could address issues inherent with the traditional diagnostic kits currently in use. SARS-CoV-2 antibody kits implementing microfluidics allow for higher sensitivity and specificity, as evident from MITOMI and other devices incorporating ELISA-based miniaturized techniques. In addition, antigen kits incorporating microfluidic technology and relevant biosensor technology resulted in the detection of viral copies of much lower numbers. Similarly, NAAT-based microfluidic technology incorporating RT-LAMP could detect viral copies of less than ten. Due to their high sensitivity and specificity, microfluidics represents the next frontier in diagnostics.

### Limitations of This Study

As this systematic review was planned and written in a short period of time, we could not find enough time to register this study. Additionally, we could not consider pieces of research that were written in languages other than English.

## Figures and Tables

**Table 1:** Microfluidic antibody detection kit for SARS-CoV-2.

Study ID	Methods	Fluid Manipulation Technique	Material	Immobilized Antigen/ Antibody/ Gene	Detected Biomolecules	Detector	Sensitivity %	Specificity %	Sample Size/ Donor/ Standard	Limit of Detection (LOD)	Detection Time	Advantages
Heggestad 2021 [21]	Microfluidic DA-D4 point-of-care test (POCT)	Pipette pump	POEGMA	S1, N, RBD	Anti-S1, anti-N, anti-RBD Abs	Fluorescent detector (D4Scope)	100% (anti-S1 & anti-RBD) 96.3% (anti-N)	100%	46 plasma samples from 31 positive patients and 41 negative samples.	NR	≤60 min	Easy to use, quantitative, high specificity and sensitivity, capable of measuring antibody kinetics and seroconversion directly from unprocessed blood or plasma, capable of detecting IP-10, low sample volume requirement, low cost.
Lin 2021 [22]	Sandwich/Competitive immune-sensors based on lateral chromatography interface	Capillary force	Polycarbonate	FMS-RBD	nAbs	Microfluidic chip fluorescence analyzer	NR	NR	182 serum samples from vaccinated participants	4–400 ng/mL (Sandwich assay) & 2.13–213 ng/mL (Competitive assay)	≤10 min	Reliable, accurate, and rapid detection of nAbs, low-cost detection.
Moncayo 2021 [23]	Semi-automated multiplexed microfluidic platform with classic multilayer soft-lithography technique	Valve pump	PDMS	S, S1, RBD, and N	Anti-S/S1/RBD/N IgG/IgM	Inverted fluorescence microscope	95	91	66 COVID positive patients	1.6 ng/mL	2.6 h	High throughput, easy to use, high sensitivity and specificity, low cost.
Swank 2020 [24]	Microfluidic nano-immunoassay platform based on MITOMI	Pneumatic valves	PDMS	His-tagged S	Anti-S IgG	Nikon ECLIPSE Ti microscope equipped with a LED Fluorescent Excitation System, a Cy3 filter set & a Hamamatsu ORCA-Flash4.0 camera	98	100	289 positive and 134 negative samples	1 nM IgG	NR	High sensitivity and specificity, 1024 samples per device, negligible reagent consumption, ultra-flow volume blood sampling
Lee 2021 [25]	Microfluidic serological assay combining nanointerstices and digitized flow control	NI driven flow force	PMMA	N	Anti-N IgG, IgM	Fluorescence reader	91.67%	100%	152 serum samples	NR	5 min	Rapid, on-site, point-of-care detection, high specificity, low cost
Funari 2020 [26]	Opto-microfluidic sensing platform with gold nanospikes based on LSRP	Syringe pump	PDMS	S	Anti-S IgG	UV–Vis spectrometer	NR	NR	NR	0.08 ng/mL	≤30 min	Easy to use, cheap, fast, promising point-of-care detection.
Gong 2021 [27]	Pulling force spinning top combined with paper-based microfluidic devices	PCBS valves	Paper	RBD	Anti-RBD IgG/IgM/IgA	Commercial smartphone	97.1 (IgA), 91.4 (IgM) & 85.7 (IgG)	100 (IgA), 92.8 (IgM) & 100 (IgG)	104 serum samples	NR	NR	Portable, high sensitivity, instrument-free, low cost
González 2021 [28]	Automated ELISA on chip	Pump	Polystyrene	S	Anti-S IgG	Microplate reader or smartphone	NR	NR	22 serum samples from 7 positive patients, 4 vaccinated and 7 negative participants	NR	NR	Low cost, reliable, rapid on-site detection, smartphone-assisted image analysis.
Liu 2020 [29]	Reciprocating-flowing immunobinding strategy	Pure water bottle pump	PDMS	N	Anti-N IgG	Commercial smartphone	NR	NR	13 patients	4.14 pg/mL	˂5 min	Rapid and efficient immunobinding capacity, reduced time consumption, low limit of detection with 100% true positive and true negative results.
Tan 2020 [30]	Microfluidic chemiluminescent ELISA technique	Capillary force	Polystyrene	S1	Anti-S1 IgG	NanoDrop™ UV-Vis spectrophotometer	NR	NR	16 convalescent patients and 3 healthy participants	10 pg/mL (LLOD)	40 min	Low time consumption, sensitive, low sample volume requirement, low detection limit
Wang 2021 [31]	Space-encoding microfluidic biochip	Pump	PDMS	N/S	Anti-N/S IgG and IgM	GenePix 4400A Microarray Scanner	NR	NR	60 serum samples	0.3 pg/mL	˂10 min (qualitative) 40 min (quantitative)	60 sample per test, fast, sensitive, Ultralow detection limit
Xu 2021 [32]	All-fiber Fresnel reflection microfluidic biosensor (FRMB)	Valve pump	Silica	S	Anti-S IgG, IgM	Photodiode detector (PD-1000)	NR	NR	6 sera spiked with anti-SARS-CoV-2 IgG/IgM	0.82 ng/mL (IgG) & 0.45 ng/mL (IgM)	7 min	Simplified structure, sensitive, label-free, easy to use, point-of-care on-site detection, reduced cost, short detection time.
Schneider 2021 [33]	Microfluidic antibody affinity profiling platform			RBD	nAbs	Biacore T200 surface plasmon resonance (SPR) system	NR	NR	42 plasma samples from seropositive individuals	NR	NR	Capable of determining the antibody affinities and concentrations of plasma antibodies
Ko 2021 [34]	Microfluidic separation of capture from detection strategy	Syringe pump	PMMA	S-RBD ligated magnetic beads	Anti-S IgG	PalmSens4 potentiostat	NR	NR	NR	~7.0 × 10^−12^ molecules of TMB (LLD)	NR	Capable of discriminating between positive patient plasma and controls, enhanced sensitivity, point-of-care detection

Abbreviation: MITOMI—mechanically induced trapping of molecular interactions; FMS—fluorescent microsphere; nAbs—neutralizing antibodies; LSPR—localized surface plasmon resonance; FB—fluoresce beads; LLOD—lower limit of detection; LLD—lowest level of detection; TMB—tetramethylbenzidine; DA—double antigen; PCBS—plastic comb binding spines; PMMA—Polymethyl methacrylate.

## Data Availability

This is review paper. All data obtained from open source.
